# Toward Sustainable and Healthy Fish Products—The Role of Feeding and Preservation Techniques

**DOI:** 10.3390/foods12162991

**Published:** 2023-08-08

**Authors:** Giorgia Antonelli, Elena Chiarello, Gianfranco Picone, Silvia Tappi, Giulia Baldi, Mattia Di Nunzio, Eleni Mente, Stelios Karapanagiotis, Phelly Vasilaki, Massimiliano Petracci, Pietro Rocculi, Alessandra Bordoni, Francesco Capozzi

**Affiliations:** 1 Department of Agricultural and Food Sciences (DISTAL), University of Bologna, Piazza Goidanich 60, 47521 Cesena, Italy; giorgia.antonelli4@unibo.it (G.A.); elena.chiarello2@unibo.it (E.C.); gianfranco.picone@unibo.it (G.P.); silvia.tappi2@unibo.it (S.T.); giulia.baldi4@unibo.it (G.B.); m.petracci@unibo.it (M.P.); pietro.rocculi3@unibo.it (P.R.); francesco.capozzi@unibo.it (F.C.); 2 Department of Food, Environmental and Nutritional Sciences (Defens), University of Milan, Via Celoria 2, 20133 Milan, Italy; mattia.dinunzio@unimi.it; 3 Laboratory of Ichthyology-Culture and Pathology of Aquatic Animals, School of Veterinary Medicine, Aristotle University of Thessaloniki, GR-54124 Thessaloniki, Greece; emente@vet.auth.gr; 4 Galaxidi Marine Farm, S.A., 33200 Galaxidi, Greece; s.karapanagiotis@galaxidimarine.farm; 5 IRIDA, Riga Fereou 60 St., GR-34600 Nea Artaki, Greece; f.vasilaki@irida.com; 6 Interdepartmental Centre for Industrial Agri-Food Research (CIRI), University of Bologna, Piazza Goidanich 60, 47521 Cesena, Italy

**Keywords:** feed, PEF, brine, in vitro digestion, lipolysis, protein hydrolysis, aquaculture, nutritional value, sea bass

## Abstract

Fish is a fundamental component of the human diet, and in the near future the proportion of aquatic foods originating from aquaculture production is expected to increase to over 56%. The sustainable growth of the aquaculture sector involves the use of new sustainable raw materials as substitutes for traditional fishmeal and fish oil ingredients, but it is crucial that the substitution maintains the nutritional value of the fish meat. In addition, the preservation of the nutritional value should be a mandatory requirement of new technologies that extend the shelf life of fish. In this context, we evaluated the impact of a newly formulated feed and three preservation treatments (brine, pulsed electric field (PEF), and PEF plus brine) on the fatty acid composition and protein and lipid digestibility of sea bass fillets. In non-digested fillets, although slightly reduced by the newly formulated feed (standard = 2.49 ± 0.14; newly formulated = 2.03 ± 0.10) the n-3/n-6 PUFA ratio indicated good nutritional value. The preservation treatments did not modify the fatty acid content and profile of non-digested fillets. Conversely, protein and lipid digestibility were not affected by the different diets but were significantly reduced by brine, with or without PEF, while PEF alone had no effect. Overall, our results indicated that the newly formulated feed containing 50% less fishmeal is a good compromise between the sustainability and nutritional value of cultivated seabass, and PEF is a promising preservation technology deserving of further study.

## 1. Introduction

The excellent nutritional value of fish is related to its content of high-value proteins, peculiar micronutrients such as iodine and selenium, and n-3 long-chain polyunsaturated fatty acids (n-3 LC-PUFAs) [1]; indeed, fish is the richest source of n-3 LC-PUFAs, especially eicosapentaenoic (EPA) and docosahexaenoic (DHA) acids, in the human diet [2].

Notwithstanding, the contribution of fish and seafood products to the diet of numerous Western countries is small [3]. This represents a nutritional concern, considering that n-3 LC-PUFAs have a variety of benefits for human health, particularly in the prevention of cardiovascular diseases [4], and their recommended intake of 250 mg/day [5] can only be achieved by increasing the consumption of fish and fish-derived products.

The n-3 LC PUFA content in fish meat, as well as in the meat of terrestrial animals, principally depends on their diet [6], and the recognition of the impact of feed on the composition of fish meat has generated a growing interest in aquaculture feeding strategies. The proportion of aquatic foods originating from aquaculture production rose from 6 percent in the 1960s to 50 percent in the 2010s; it was estimated to have further increased to 56 percent by 2020 [3] and it is expected to continue growing to meet future demand. In aquaculture feed, fishmeal and fish oil are the primary sources of protein and n-3 LC PUFAs. Unfortunately, they are also limiting factors for the growing fish farming industry [7,8]. The sustainable growth of the aquaculture sector involves the use of new sustainable raw materials as substitutes for traditional fishmeal and fish oil ingredients, but it is crucial that the substitutes maintain the nutritional value of the fish meat. Fish performance, health status, and final product quality may be significantly affected when substituting dietary fishmeal with alternative ingredients in aquaculture diets [7,8]. Changing the diet of farmed fish to a more plant-based one could result in a decrease in n-3 LC PUFAs and an increase in n-6 fatty acids, thus limiting the positive effect of fish consumption in humans [9]. Therefore, it is necessary to study different formulations that are on the one hand more sustainable but on the other hand do not reduce the nutritional value of the fish. From this perspective, krill meal is considered a good candidate for replacing fishmeal.

Innovative approaches are required to address an additional issue pertaining to the consumption of fish, which is its elevated susceptibility to spoilage. The pros and cons of the traditional preservation technology approaches for fish have been recently revised by Ali et al. [10]. Refrigeration is probably one of the most used methods for fish preservation, coupled or not with modified atmosphere packaging, but even under these conditions, fish has a very short shelf life. Fish shelf life can be extended by freezing, but freezing/thawing largely alters the fish’s fresh-like characteristics. Drying is another preservation technique, widely used around the world, using solar energy, hot air, or, more recently, microwaves, to remove water and increase shelf life. Depending on the method used for drying, fat oxidation often occurs during the storage of dried fish products. Canning is the second most popular method of preserving fish for human consumption and results in a very long shelf life (1–5 years). However, the sterilization process has been shown to negatively affect the quality characteristics of fish, particularly lean fish. Moreover, thermal treatment can lead to changes in nutritional quality, particularly changes in fatty acid distribution and reduction in the n-3/n-6 ratio [11]. In the past, salting and brining was the most common way to preserve virtually any type of meat or fish; today it is known that the excessive presence of salt/sodium in food and in the diet increases the risk of hypertension and cardiovascular diseases. Therefore, the seafood industry is looking for alternatives and non-thermal food processing technologies seem promising for shelf life extension while maintaining good sensory and nutritional characteristics of the fish. Among non-thermal technologies, pulsed electric fields (PEFs) have gained importance in food processing. PEF is an emerging technology that involves subjecting the food product to short (few µs), high-voltage electric pulses, causing an effect known as ‘electroporation’ of the cell membranes. The process produces modest thermal increases without any effect on the product and it was reported to have a good impact on the microstructure of muscle foods, without affecting physical, organoleptic, and functional characteristics [12].

To speed up innovation in the fishing industry, the effects of different diets on the composition of fish meat have been the subject of many studies [13,14], and the evaluation of microbial inactivation and sensory quality of fish products after different preservation treatments, alone and in combination, has been addressed in recent works [15,16,17]. However, the effect of newly formulated feeding strategies and postmortem preservation technologies on the nutritional quality and digestibility of fish meat has rarely been addressed in the same study. This is crucial, as feeding can influence the content of n-3 LC PUFAs and preservation technologies can modify the digestibility of fish meat. 

The main purpose of this study was to provide information to the fishing industry on the strategies to be adopted to increase the sustainability of aquaculture and to reduce the perishability of fish while maintaining its nutritional value. Thus, we evaluated the impact on the fatty acid (FA) composition and on the protein and lipid digestibility of sea bass (*Dicentrarchus labrax*) fillets of two feeding strategies (standard and newly formulated, containing 50% less fishmeal) and three preservation treatments (brine, pulsed electric field (PEF), and PEF plus brine).

## 2. Materials and Methods

### 2.1. Materials

Unless specified, the chemicals and solvents were of the highest analytical grade and purchased from Merck (Darmstadt, Germany) and Sigma-Aldrich (St. Louis, MO, USA).

### 2.2. Farming Trial

All the procedures were conducted following the European Union guidelines for the ethical care and handling of animals under experimental conditions (2010/63/EU) and in accordance with the Animal Protocol Review Committee of the University of Thessaly (EL-43BIO). The farming trial was carried out at the Galaxidi fish farm in Greece where a standard commercial and a newly formulated, potentially organic diet were tested in sea bass populations in triplicate for 12 months. Approximately 2000 sea bass (*Dicentrarchus labrax*) of an initial weight of 60 g were stocked in six net cages of 60 m^3^ capacity and fed the standard or newly formulated feed (three cages/dietary treatment) produced by IRIDA (Nea Artaki Evia, Greece). The standard feed had a semi-confidential formula, while the newly formulated feed was formulated to contain fishmeal replacers such as squid and krill meals (Table 1).

The two diets were nutritionally balanced with other ingredients commonly used in aquafeeds as well as with macro and micronutrients. The proximate composition of amino acids and fatty acids is shown in Table 2 and Table 3, respectively.

Sea bass fish were fed twice per day an average of 0.86% of their body weight (b.w.) depending on the water rearing temperature and fish b.w. The environmental parameters, feed consumption, and mortality were recorded daily, and the fish were weighed at different time points to calculate the fish growth parameters. Specific growth rate (SGR), feed conversion ratio (FCR), and specific feeding rate (SFR) were calculated according to the following formulas:

SGR (% day) = %growth/day = 100 × (lnWfin − lnWin)/d

FCR (g/g) = Feed Intake (g)/Weight gain (g)

SFR (% day) = food eaten (g)/day/fish weight × 100

where Wfin is the final mean weight (g), Win is the initial mean weight (g), and d is the duration of feeding (days).

### 2.3. Histopathological Examination

At the end of the feeding trial, six sea bass from each dietary group were taken for histopathological examination. After being euthanized, the fish were immediately placed on ice. Samples of the liver and anterior gut were taken from each fish, fixed in 4% formaldehyde for 24 h at 4 °C, dehydrated in a graded series of ethanol, immersed in xylol, and embedded in paraffin wax. Sections of 5–7 μm were mounted, deparaffinized, rehydrated, stained with hematoxylin–eosin, mounted with Cristal/Mount, and examined for alterations with a microscope (Axiostar plus Carl Zeiss Light Microscopy, Carl Zeiss Ltd., Gottingen, Germany) under a total magnification of 50×, 100×, 400×, and 1000×.

### 2.4. Sample Preparation and Fish Processing

At the end of the experiment, a total of 20 fish per dietary treatment were beheaded, gutted, skinned, and filleted. The superior part of each fillet was cut into square samples (2 × 2 cm, average weight 7.5 ± 0.6 g, 2 pieces for each fillet) and underwent different treatments: (1) brining; (2) PEF; and (3) PEF + brining (20 samples/treatment) at the Department of Agricultural and Food Sciences (DISTAL) of the University of Bologna. 

PEF treatment was performed using a lab-scale PEF unit delivering a maximum output current and voltage of 60 A and 8 kV, respectively (Mod. S-P7500, Alintel, Bologna, Italy). The generator provided monopolar rectangular-shaped pulses and adjustable pulse duration (5–20 µs), pulse frequency (50–500 Hz), and total treatment time (1–600 s). The treatment chamber (50 mm length × 50 mm width × 50 mm height) consisted of two parallel stainless-steel electrodes (3 mm thick) with a 47 mm fixed gap. The output voltage and current were monitored using a PC oscilloscope (Picoscope 2204a, Pico Technology, St Neots, UK). The PEF treatment parameters were as follows: voltage 0.6 kV/cm; frequency 100 Hz; pulse width 10 µs; repetition time 10 ms; pulse number 1000; treatment time 10 s. 

The brining procedure was by immersion of samples in a 10% NaCl solution, prepared using edible NaCl purchased from a local market. The combined treatment was obtained by subjecting the samples to PEF and subsequently to brine. After 5 days, the samples were removed from the solution, blotted with absorbent paper, and evaluated immediately for mass transfer parameters (weight gain, water, and NaCl content). 

All samples were stored individually at −20 °C until further analyses.

### 2.5. Mass Transfer Parameters

The weight of each sample was measured with an analytical balance (mod. Europe 8000, Gibertini, Milan, Italy). Water content was determined by drying a 2 g sample at 105 °C for 24 h, according to the official method AOAC, 2005. Salt (NaCl) content was determined by titration according to AOAC 976.18 (1995). Each determination was carried out on 5 samples.

### 2.6. Protein Solubility in Non-Digested Samples

Total protein solubility was measured according to Sotelo et al. [18] with slight modifications. In detail, one gram of muscle tissue was homogenized by UltraTurrax T25 Basic (IKA-Werke, Staufen im Breisgau, Germany) at 13,000 rpm for 30 s in 10 mL of ice-cold 5% NaCl and 20 mM NaHCO_3_ solution (pH 7.00) and then centrifuged at 13,000× *g* for 20 min at refrigerated conditions. The resulting supernatant, consisting of the sarcoplasmic proteins, was separated, while 10 mL of ice-cold 1% NaCl and 20 mM NaHCO_3_ solution (pH 7.00) was added to the pellets. The samples were then centrifuged again under the above-reported conditions, and the resulting supernatant containing the myofibrillar proteins was properly separated. After appropriate dilution, the supernatants were used for protein quantification by Bradford’s method using bovine serum albumin as standard [19]. Total protein solubility (mg/g) was calculated as the sum of the concentrations of both myofibrillar and sarcoplasmic fractions.

### 2.7. In Vitro Digestion

In vitro static digestion was performed according to the INFOGEST protocol [20]. Briefly, 7 g of each sample was chopped to simulate chewing and then mixed with 5.6 mL of simulated salivary fluid (SSF) containing 15.1 mM KCl, 3.7 mM KH_2_PO_4_, 13.6 mM NaHCO_3_, 0.15 mM MgCl_2_(H_2_O)_6_, 0.06 mM (NH_4_)_2_CO_3_, pH 7, and 35 µL of calcium chloride 0.3 M and 1.365 mL of distilled water for two minutes at 37 °C. Then, 11.2 mL of simulated gastric fluid (SGF) (6.9 mM KCl, 0.9 mM KH_2_PO_4_, 25 mM NaHCO_3_, 47.2 mM NaCl, 0.12 mM MgCl_2_(H_2_O)_6_, 0.5 mM (NH_4_)_2_CO_3_, pH 3), 2.69 mL pepsin (f. c. 2000 U/mL), and 7 µL of 0.3 M calcium chloride were added. The pH was lowered to 3 using HCl 37% and the flask was kept stirring at 37 °C for 2 h in a thermostatic water bath. At the end of the gastric phase, 12.4 mL of simulated intestinal fluid (SIF) containing 6.8 mM KCl, 0.8 mM KH_2_PO_4_, 85 mM NaHCO_3_, 38.4 mM NaCl, 0.33 mM MgCl_2_(H_2_O)_6_, 10 mL pancreatin (f. c. 100 U/mL), 3.5 mL bile (f. c. 10 mM), 1.94 mL water, and 56 µL of 0.3 M calcium chloride were added and the pH was raised to 7 using NaOH 35%. The flask was kept stirring for two hours at 37 °C. At the end of the duodenal phase, the digesta were collected and the enzymes were inactivated by lowering the pH to 3 and then raising it back to pH 7. The samples were centrifuged at 4500× *g* for 10 min at 4 °C and then stored at −20 °C until further analysis.

### 2.8. Fatty Acid Composition and Content of Sea Bass Fillets

Total lipids were extracted from non-digested and digested sea bass fillets according to Bligh and Dyer [21] with slight modifications. Briefly, 6 mL of methanol, 3 mL of chloroform, and 2.4 mL of distilled water were sequentially added to 0.1 g of non-digested or 0.8 mL of digested sample, each addition followed by homogenization by UltraTurrax T10 Basic (IKA-Werke, Staufen im Breisgau, Germany) for 30 s (non-digested samples) or mixed with magnetic stirring (digested samples). Then, 3 mL of chloroform and 3 mL of distilled water were added, and the solution was homogenized with UltraTurrax or mixed with magnetic stirring after every addition. The chloroform layer was collected in a test tube, with 1 mg of internal standard added (pentadecanoic acid), and dried under nitrogen infusion. The methylation of fatty acids was performed by adding 500 µL of hydrogen chloride solution 0.5 M in methanol (Sigma-Aldrich, Milan, Italy, 07607) at 100 °C for 1 h. At the end of the methylation step, 2 mL of hexane and 2 mL of distilled water were sequentially added [22]. The hexane layer was transferred in a test tube and dried under nitrogen infusion. The resulting fatty acid methyl esters (FAMEs) were suspended in 100 µL of hexane. The analysis of FAMEs was performed by fast GC (GC-2030, Shimadzu, Kyoto, Japan) equipped with a MEGA-10 capillary column (30 mt, 0.2 mm ID, 0.2 μm film thickness) with a programmed temperature gradient (50–250 °C, 10 °C/min). The peaks were identified based on their retention time, which was predetermined using a standard mix solution (Supelco, Milan, Italy, CRM47885) and quantified using Lab Solution Software version 5.99 (Shimadzu, Kyoto, Japan) [23].

### 2.9. Evaluation of Protein Hydrolysis in In Vitro Digested Sea Bass Fillets

Digested samples were centrifuged at 50,000× *g* for 20 min at 4 °C and then filtered on a 0.22 µm syringe filter. Protein concentration was assessed spectrophotometrically by o-phthaldialdehyde (OPA) assay [24], measuring the absorbance at 280 nm [23] using L-glutamic acid and non-fat dry milk as standards, respectively. The protein content from the enzymes added during in vitro digestion was subtracted, and the values were standardized for the dilution factor due to the addition of digestive fluids.

### 2.10. ^1^H Nuclear Magnetic Resonance (NMR) for In Vitro Digested Samples

Digested sea bass samples were prepared according to Picone et al. [25]. Briefly, samples were thawed and centrifuged at 2300× *g* for 10 min at 4 °C. Then, 750 µL of supernatant was taken and added to 120 µL of 100 mM phosphate buffer with 10 mM sodium trimethylsilylpropanesulfonate (DSS). The pH value was adjusted to 7.00 ± 0.05, and then the samples were centrifuged again at 2300× *g* for 10 min at 4 °C. HR-NMR spectra were recorded on a Bruker US+ Avance III spectrometer operating at 600 MHz, equipped with a BBI-z probe and a cooled 24-sample storage for acquisition automation (Bruker BioSpin, Karlsruhe, Germany). 

The progression of in vitro digestion was evaluated in five different spectral regions, collecting signals from hydrogen atoms located on hydrophobic amino acids (0.20–2.00 ppm), hydrophilic amino acids (2.00–3.00 ppm), total amino acids—α protons (3.20–4.70 ppm), aromatic amino acids (6.40–7.70 ppm), and total soluble proteins (7.70–9.00 ppm). The integrals of these areas were calculated after spectra normalization on the inner reference standard (DSS).

### 2.11. Statistical Analysis

Statistical analysis was performed using one-way ANOVA with Tukey’s post hoc test to compare the different preservation treatments or using Student’s *t*-test to compare the two different feeds, assuming *p* < 0.05 as significant.

## 3. Results and Discussion

### 3.1. Effects of Feeding

**Figure 1:**
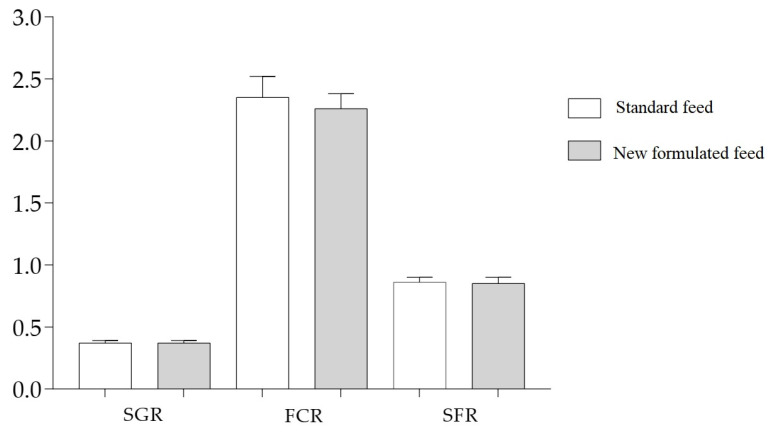
Specific growth rate (SGR, %/day), feed conversion rate (FCR, g), and specific feeding rate (SFR, %/day) in the two dietary groups (standard and newly formulated). Data are mean ± SD of triplicate determinations. Statistical analysis was performed using Student’s *t*-test assuming *p* < 0.05 as significant (n.s.).

During the trial, fish body weight increased from 60 to 380 g irrespective of the dietary treatment. Average SGR, FCR, and SFR had similar values for both dietary treatments (Figure 1).

In both fish groups, the histopathological examination of the liver showed mild and moderate lipid accumulation. The anterior gut showed no histopathological lesions (Supplementary Figure S1). One fish showed white blood cell infiltration (Supplementary Figure S1).

**Figure 2:**
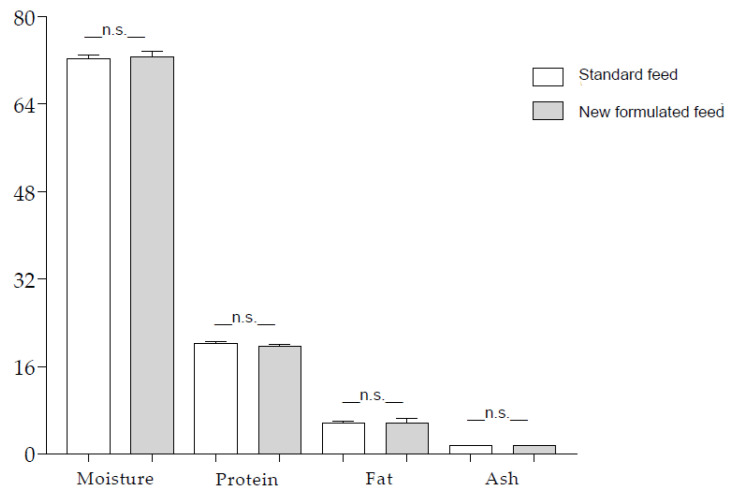
Composition of fillets from sea bass fed the standard and newly formulated diets. Data are mean ± SD of triplicate determination and are expressed as g/100 g. Statistical analysis was performed using Student’s *t*-test, assuming *p* < 0.05 as significant (n.s.).

Regardless of the feed, non-treated (NT) fillets had a similar composition (Figure 2) and total content of FAs (standard diet = 2212.96 ± 406.91 mg/100 g; newly formulated diet = 2939.89 ± 275.27 mg/100 g; n.s.), confirming that dietary lipid sources did not affect lipid deposition in sea bass muscle [26].

The most abundant FAs were oleic (18:1 n-9, OA), palmitic (16:0, PA), and DHA (22:6 n-3) (Table 4), as reported by Baki et al. [27] in cultivated sea bass.

The differences observed in the fatty acid profiles of fillets did not reflect the differences in the FA content of the feed. Apparently, our results contradict those obtained by Verge-Mèrida et al. [28], who reported diet-related modifications in the FA composition of tissues from sea bass fed different diets. It should anyway be considered that the diets used in that study were very different from each other, with n-3 PUFA content ranging from 31.55 to 11.01% and MUFA content from 52.84 to 24.45%, while the diets used in our study did not have a very different FA profile. Verge-Mèrida et al. [28] observed differences in the FA composition of flesh only when comparing extreme dietary contents, and the differences in MUFA and PUFA composition were more clearly reflected in the FA profile of perivisceral fat than in the muscle. Similar results were observed by Izquierdo et al. [26].

Despite the high intake of cetoleic acid (C22:1 n-11, CA) in both diets, the content of this fatty acid in fillets was negligible, according to Baki et al. [22]. This could be related to the high CA utilization by sea bass as an energy source, as already reported in salmon and trout [24]. CA, which was twice as high in the newly formulated feed as in the standard feed, was reported to affect the ability of the fish liver to convert α-linolenic acid (C18:3n-3, ALA) to DHA via the potential stimulation of peroxisomal β-oxidation [29]. In our study, if any increase in ALA conversion occurred in sea bass liver it did not lead to an increased n-3 LC-PUFA content in fillets. 

The different feeds caused a significant modification in the rate of n-3/n-6 PUFAs (2.49 ± 0.14 and 2.03 ± 0.10 in fillets from sea bass fed the standard and newly formulated diet, respectively; *p* < 0.05). Since the proportion of n-3 to n-6 FAs in a diet may have metabolic consequences [30], the rate of n-3/n-6 PUFAs is considered an indicator of food quality in terms of nutrition, with rates higher than 1 indicating good nutritional value. Overall, although the different diets caused a modification in the FA composition of non-treated fillets, the nutritional value of the sea bass flesh was still high.

### 3.2. Effects of Preservation Technologies on FA Content and Composition

Regardless of the dietary treatment, brining and PEF plus brining significantly decreased the total content of FAs compared to NT fillets, while PEF treatment alone did not cause any modification (Table 5).

Since the same quantity of sample (100 mg) was used for all lipid extractions, we assume that the decrease in the FA content of fillets after brining and PEF + brining was mainly the consequence of the increase in water content and weight due to the treatment (Table 6). In addition, it is conceivable that the different preservation technologies modified the food matrix, making the lipid extraction differently exhaustive.

The total SFA, MUFA, and PUFA percentage contents of sea bass fillets were similar regardless of the processing treatment, except for a slight decrease in MUFAs in fillets from fish fed the standard diet. The percentage FA compositions of NT and treated fillets from sea bass fed the different diets are given in Supplementary Table S1.

### 3.3. Lipid Hydrolysis after In Vitro Digestion

In NT fillets, the different feeds did not impact the quantity of FAs released from the matrix after in vitro digestion (Table 7). To detect a higher amount of FAs in digested samples than in corresponding undigested ones is not surprising. In fact, in vitro digestion disintegrates the food matrix, facilitating the extraction of the components. Therefore, the quantity of FAs in the digests could be higher since the lipid extraction was applied to a less compact sample. To confirm this hypothesis, in a previous work [31] we showed that in vitro digestion is a more exhaustive procedure for extracting polyphenols from a solid food matrix. In addition, to obtain comparable results, we performed the fat extraction in all samples with the Bligh and Dyer method, which is widely accepted for the extraction of fat components from highly aqueous systems, such as small intestinal digests [32].

The different processing modulated the release of FAs during digestion, which was reduced by the brine and, in the case of sea bass fed the standard diet, by the PEF plus brine (Table 7).

A modulation of the kinetics of the fatty acid release from the food matrix during in vitro digestion by different salt concentrations was already observed in Parmigiano Reggiano cheese [23], and Nieva-Echevarría et al. previously observed a high degree of lipolysis during in vitro digestion of unsalted sea bass [33]. The less effective lipolysis in brined fillets could be due to the increase in the NaCl:moisture ratio, which could decrease the amount of water available for triglyceride hydrolysis [34]. PEF alone had no impact on the release of FAs during digestion. 

In fillets from sea bass fed the standard diet, the different processing treatments did not modify the total percentage content of released SFAs, MUFAs, and n-6 and n-3 PUFAs. In sea bass fed the newly formulated diet, a lower release of SFAs after brining and a higher release of MUFAs after PEF treatment was observed compared to NT samples. The percentage composition of FAs released from the matrix after in vitro digestion is given in Supplementary Table S2.

### 3.4. Protein Hydrolysis after In Vitro Digestion

The rate of protein hydrolysis after in vitro digestion was evaluated in the duodenal digesta with two different spectrophotometric methods, OPA and absorbance at 280 nm. Although each technique has some drawbacks, the comparative application on similar substrates still provides useful information, as under- or over-estimation are parallel in all samples. The spectrophotometric reading at 280 nm accounts for the fraction of soluble amino acids, peptides, and proteins containing aromatic side chains [35]. The method is reliable if the amino acid composition of fillet proteins is equal to that of one of the standard proteins, a condition that is not always maintained during digestion. The OPA assay is sensitive to the free amino end of amino acids and peptides [36]. Thus, the method is strongly dependent on the level of protein hydrolysis; keeping the quantity of hydrolyzed proteins constant, the smaller the fragments, the higher the response. 

In NT samples, protein hydrolysis detected by the OPA method was similar in standard and newly formulated diet groups while a significant difference was detected by measuring it as absorbance at 280 nm (*p* < 0.02). 

Comparing the different treatments, a significant decrease in protein hydrolysis was detected in fillets subjected to brining and PEF + brining, independent of the sea bass feed and the detection method used (Table 8).

To further investigate the impact of feeding and processing on fillet digestion, NMR spectroscopy was applied to the same samples analyzed with the spectrophotometric assays. The advantage of using this further technique is associated with its universal detection capability, without the requirement of an external standard to calculate an instrumental response factor, provided that the molecules under investigation contain at least one atom of hydrogen and are soluble in the solvent of the sample. All molecules released by digestion satisfy these requirements, including amino acids, peptides, and larger soluble fragments of proteins. Thus, the area of diagnostic signals in specific regions of the NMR spectrum is directly proportional to the concentration of hydrogen atoms belonging to the molecule to be quantified (either single amino acids, short peptides, or small or large protein fragments). As only the soluble molecules are detected, the NMR technique provides the condition necessary to evaluate the accessibility of nutrients upon digestion. Table 9 reports the results of the NMR spectroscopy analysis carried out to evaluate protein hydrolysis after in vitro digestion in NT and treated samples.

An overall 30% reduction in protein digestion was observed in brined fillets (with or without PEF pre-phase) compared to the NT ones, regardless of the fish diet. Since the decrease was of the same magnitude in all NMR spectral regions, it is argued that brining did not affect the digestion profile, but it caused a generalized decrease in overall protein digestibility. 

We speculate that the observed decrease in protein digestibility was caused by the so-called “salting-out”. At low salt concentrations, proteins are surrounded by salt counterions (ions of opposite net charge), and this results in decreased electrostatic free energy of the protein and increased activity of the solvent, which in turn leads to increased solubility (salting-in). The abundance of the salt ions decreases the solvating power of salt ions, resulting in a decrease in the solubility of the proteins and precipitation results (salting-out) [37]. In our experimental conditions, the initial salt content in the sea bass fillets was 0.00%. After brining, it increased to more than 5%, without significant differences between fillets from fish fed the standard and newly formulated diets (Table 6). In the standard feed group, pre-treatment with PEF did not change salt uptake during brining, while it increased the salt content to 6.7% in the newly formulated feed group. Protein solubility in non-digested fillets (Table 10) confirmed that at these NaCl concentrations the “salting-out” occurred, thus explaining the decreased protein digestibility after the brining and brining plus PEF treatments.

To summarize, the newly formulated feed tested in our trial appears promising as a means to increase sustainable aquaculture. 

## 4. Conclusions

The expansion of aquaculture production has been accompanied by the need for rapid growth in feed production. The challenge facing the aquaculture industry is to identify economically viable and environmentally friendly alternatives to fishmeal and fish oil on which many fish feeds are largely based. The formulation of more sustainable feed is extremely important for fish farming as there is a need to limit the growing demand for fishmeal and fish oil [31]. Feed ingredients with low environmental effects and a low carbon footprint could promote a successful and sustainable aquaculture strategy if they have no negative impact on the nutritional value of the fish meat. From this perspective, krill and squid meals are considered good candidates for replacing fishmeal [38,39,40].

In this study, the use of a newly formulated feed, in which 50% of the fishmeal was replaced by low-trophic-level organisms, yeast protein, and plant ingredients appeared a good compromise as it had no negative effects on the nutritional value of the sea bass fillets, which was evaluated considering not only on the chemical composition but also protein and lipid digestibility. Digestibility is an accurate indicator of nutritional value as digestion produces the mass of bioaccessible molecules (fatty acids from lipids and small peptides/amino acids from proteins) which can be absorbed by enterocytes, enter the human body, and effectively perform their functions. Since nutrients in a food are not totally bioaccessible, the chemical composition does not exactly reflect the nutritional value. 

To reduce the burden on the wild ecosystem of the use of krill and squid meal, the krill meal used in the newly formulated feed came from one of the most sustainable fisheries in the world, which harvests krill in Antarctica’s Area 48 only. Krill meal from Antarctica’s Area 48 is certified by the Marine Stewardship Council (MSC) for being 100% sustainable and traceable. Squid meal was purchased from the first squid fishery in the world that achieved MSC certification as a sustainable and well-managed stock.

Our results highlight that the accurate choice of ingredients reduces the environmental cost of feed without affecting the nutritional value of the fish. 

Furthermore, we have highlighted that, although it is important to counteract the high perishability of fish, the possible modifications in the nutritional value determined by the preservation treatments usually applied to prolong the shelf life must be carefully evaluated. In our experimental conditions, PEF treatment did not have any negative impact on fish digestibility and experiments are underway to evaluate the effect of this non-thermal technology on product shelf life. However, for a possible implementation of this technology, possible modifications in the sensorial properties of the products should be assessed since they might affect consumer acceptability. Although it is reported that PEF does not alter the organoleptic characteristics of the product [17], further studies are needed to evaluate the sensory characteristics of PEF-treated fillets. By contrast, brining (with or without PEF pre-phase) significantly reduced lipid and protein hydrolysis during in vitro digestion and from a nutritional point of view cannot be considered a suitable method to counteract the high perishability of fish, even in consideration of the increased sodium content. 

Health authorities’ advice has encouraged consumers to eat more fish, and global fish consumption has increased by more than 100%. To make this increase consistent with improving human health and being environmentally friendly, strong cooperation between different stakeholders is required. The results reported here represent a further step toward strategies that could innovate the fishing industry by allowing the production of fish products with high nutritional value, reduced perishability, and lower environmental impact.

## Figures and Tables

**Table 1:** Ingredients (g/100 g dry weight) of the standard and newly formulated feed.

Ingredient (%)	Standard Feed	Newly Formulated Feed
Fishmeal	51	25
Soya bean meal	23.5	-
Fermented soya	-	13
Wheat gluten	-	5
Wheat	15	12.45
Fish oil	10	10.8
Pea protein	-	10
Krill meal	-	6
Squid meal	-	6
Yeast	-	5
Corn gluten meal	-	5
Monocalcium phosphate	-	1.2
Synergen	-	0.05
Premix	0.5	0.5

**Table 2:** Proximate composition of the standard and newly formulated feed.

	Standard Feed	Newly Formulated Feed
Moisture (%)	4.97 ± 0.04	8.13 ± 0.01 *
Dry Weight (%)	95.03 ± 0.04	91.86 ± 0.15 *
Proteins (%)	46.56 ± 0.12	47.07 ± 0.21
Lipids (%)	14.72 ± 0.08	14.93 ± 0.32
Ash (%)	12.21 ± 0.03	7.74 ± 0.03 *
Energy (Kj/g)	20.90 ± 0.04	22.29 ± 0.03 *

Data are mean ± SD of triplicate determination. Statistical analysis was performed using Student’s *t*-test (* *p* < 0.0001).

**Table 3:** Amino acid and fatty acid composition of the standard and newly formulated feed.

Amino Acid (%)	Standard Feed	Newly Formulated Feed	Fatty Acid (%)	Standard Feed	Newly Formulated Feed
Aspartic acid	7.7	10.7	14:0	3.58	4.68
Glutamic acid	12.2	14.9	16:0	14.29	15.25
Serine	5.4	6.1	16:1 n-7	4.07	4.56
Glycine	8	6.6	18:0	3.49	3.16
Histidine	1.5	1.4	18:1 n-9	28.29	17.68
Arginine	8.3	5.9	18:1 n-7	3.27	2.71
Threonine	4	4.5	18:2 n-6	12.71	10.69
Alanine	7.2	6	18:3 n-3	3.99	2.89
Proline	7.3	8.2	18:4 n-3	1.38	2.21
Tyrosine	3.2	3.3	20:1 n-9	4.43	7.03
Valine	5.6	5.3	20:2 n-6	0.56	0.36
Methionine	2.1	1.3	20:4 n-6	0.52	0.60
Isoleucine	4.5	4.7	20:5 n-3	4.94	5.59
Leucine	8.6	9.5	22:1 n-11	4.72	9.87
Phenylalanine	5.2	5.4	22:1 n-9	0.68	1.00
Lysine	9.5	6.4	22:6 n-3	5.83	8.01
Total EAAs	52.5	47.7	24:1 n-9	0.60	0.88
Total NEAAs	47.8	52.5	Total SFAs	21.36	23.09
			Total MUFAs	46.06	43.73
			Total n-6 PUFAs	13.79	11.65
			Total n-3 PUFAs	16.16	18.70

Data are reported as percentages. Fatty acids lower than 0.5% are not reported. EAAs = essential amino acids; NEAAs = non-essential amino acids; SFAs = saturated fatty acids; MUFAs = monounsaturated fatty acids; PUFAs = polyunsaturated fatty acids.

**Table 4:** Percent fatty acid composition of NT fillets of sea bass fed the different diets.

	Standard Feed	Newly Formulated Feed
14:0	3.31 ± 0.07	3.12 ± 0.12
16:0	19.78 ± 0.44	20.37 ± 0.21
16:1 n-7	3.98 ± 0.28	4.42 ± 0.10
18:0	4.15 ± 0.26	4.47 ± 0.15
18:1 n-9	25.82 ± 0.78	30.14 ± 0.37 ***
18:2 n-6	9.69 ± 0.05	9.88 ± 0.24
18:3 n-3	2.40 ± 0.10	2.44 ± 0.06
20:1 n-9	5.69 ± 0.13	4.85 ± 0.19 **
20:4 n-6	0.98 ± 0.01	0.91 ± 0.03 *
20:5 n-3	6.78 ± 0.47	5.69 ± 0.31 *
22:5 n-3	1.49 ± 0.04	1.35 ± 0.04 *
22:6 n-3	15.92 ± 0.73	12.36 ± 0.50 **
ΣSFA	27.25 ± 0.71	27.97 ± 0.19
ΣMUFA	35.48 ± 0.95	39.41 ± 0.58 **
ΣPUFA n-3	26.60 ± 1.21	21.84 ± 0.82 **
ΣPUFA n-6	10.67 ± 0.05	10.78 ± 0.22

Data are expressed as mol/100 mol and are means ± SD of three biological replicates. Statistical analysis was performed using Student’s *t*-test, assuming *p* < 0.05 as significant (* *p* < 0.05; ** *p* < 0.01; *** *p* < 0.001). NT = non-treated.

**Table 5:** Total content of fatty acids in NT and treated fillets of sea bass fed the different diets.

	NT	Brine	PEF	PEF + Brine
Standard diet	2212.96±406.9 ^a^	760.98±47.91 ^b^	1883.28±86.18 ^a^	1308.31± 1.84 ^b^
Newly formulated	2939.89±275.27 ^a^	1501.29±367.04 ^b,^*	2262.51±626.41 ^a,b^	1520.34±214.06 ^b^

Data are expressed as mg FAMEs/100 g sample and are means ± SD of 3 biological replicates. Statistical analysis was performed using one-way ANOVA (standard feed *p* < 0.0001; innovative feed *p* = 0.0066) with Tukey’s post hoc test to compare the different treatments in fish fed the same diet (different letters in the same row indicate statistical significance, at least *p* < 0.05) and using Student’s *t*-test to compare the effect of the same treatment in fish fed the different diets (* *p* < 0.05). NT = non-treated; PEF = pulsed electric field.

**Table 6:** Weight, water content, and salt content of NT and treated fillets of sea bass fed the different diets.

Weight (g/Square Sample)					
	NT	Brine	PEF	PEF + Brine	*p*-value
Standard diet	5.51 ± 0.76 ^a^	7.79 ± 0.58 ^b^	6.11 ± 0.41 ^a^	8.20 ± 0.43 ^b^	<0.001
Newly formulated	5.75 ± 0.68 ^a^	7.99 ± 1.08 ^b^	6.49 ± 0.49 ^a^	8.85 ± 0.73 ^b^	<0.001
Water content (g/100 g)					
	NT	Brine	PEF	PEF + Brine	*p*-value
Standard diet	71.74 ± 3.53 ^a^	78.32 ± 1.87 ^c^	73.88 ± 2.24 ^a,b^	77.90 ± 1.13 ^b,c^	<0.001
Newly formulated	71.82 ± 3.02 ^a^	74.62 ± 2.06 ^a,b,^*	71.86 ± 3.09 ^a^	76.46 ± 1.42 ^b^	0.0245
NaCl content (g/100 g)					
	NT	Brine	PEF	PEF + Brine	*p*-value
Standard diet	n.d.	5.18 ± 1.08 ^a^	n.d.	4.91 ± 0.88 ^a^	0.668
Newly formulated	n.d.	5.94 ± 0.52 ^a^	n.d.	6.71 ± 0.65 ^b,^*	0.0048

Data are means ± SD of 5 biological replicates. Statistical analysis was performed using one-way ANOVA with Tukey’s post hoc test to compare the different treatments in fish fed the same diet (different letters in the same row indicate statistical significance, at least *p* < 0.05) and using Student’s *t*-test to compare the effect of the same treatment in fish fed the different diets (* at least *p* < 0.05). NT = non-treated; PEF = pulsed electric field; n.d. = not detected.

**Table 7:** Release of FAs after in vitro digestion of NT and treated fillets of sea bass fed the different diets.

	NT	Brine	PEF	PEF + Brine
Standard feed	3734.01 ± 230.80 ^a^	1599.94 ± 406.35 ^b^	3340.49 ± 617.90 ^a^	1409.68 ± 216.85 ^b^
Newly formulated	3912.22 ± 622.22 ^a^	2616.81 ± 28.94 ^b^	3227.62 ± 177.80 ^a^	3357.20 ± 30.90 ^a^

Data are expressed as mg FAMEs/100 g sample and are means ± SD of 3 biological replicates. Statistical analysis was performed using one-way ANOVA with Tukey’s post hoc test. Different letters in the same row indicate statistical significance (at least *p* < 0.05). NT = non-treated; PEF = pulsed electric field.

**Table 8:** Protein content in NT and treated digested samples.

Standard Feed					
	NT	Brine	PEF	PEF + Brine	*p*-value
A 280 nm	11.25 ± 0.93 ^a^	7.77 ± 0.45 ^b^	11.82 ± 0.51 ^a^	7.15 ± 0.95 ^b^	<0.0001
OPA	22.83 ± 4.21 ^a^	12.97 ± 0.34 ^b^	25.41 ± 1.33 ^a^	14.14 ± 2.54 ^b^	0.0012
Newly formulated feed					
	NT	Brine	PEF	PEF + Brine	*p*-value
A 280 nm	14.42 ± 1.30 ^a^	7.50 ± 0.22 ^c^	12.38 ± 0.48 ^a,b^	10.17 ± 1.05 ^b^	<0.0001
OPA	26.71 ± 3.08 ^a^	13.21 ± 1.42 ^b^	31.16 ± 2.82 ^a^	14.12 ± 0.49 ^b^	<0.0001

Data are expressed as mg protein/100 g digested sample and are means ± SD of 3 biological replicates. Statistical analysis was performed using one-way ANOVA with Tukey’s post hoc test. Different letters in the same row indicate statistical significance (at least *p* < 0.05). NT = non-treated; PEF = pulsed electric field.

**Table 9:** Integral values of the 5 main NMR spectrum areas. Data are means ± SD of 3 biological replicates.

Standard Feed					
	NT	Brine	PEF	PEF + Brine	*p*-value
Hydrophobic amino acids (0.20–2.00 ppm)	151.28 ± 7.39 ^a^	107.71 ± 14.27 ^a,b^	144.79 ± 18.84 ^a^	100.52 ± 22.37 ^b^	0.0121
Hydrophilic amino acids (2.00–3.00 ppm)	53.14 ± 3.18 ^a^	35.80 ± 4.80 ^b^	51.28 ± 6.79 ^a^	32.97 ± 7.54 ^b^	0.0052
Total amino acids (α protons) (3.20–4.70 ppm)	98.93 ± 3.84 ^a^	68.82 ± 8.07 ^b^	94.98 ± 9.40 ^a^	64.20 ± 13.46 ^b^	0.0034
Aromatic amino acids (6.40–7.70 ppm)	13.72 ± 0.6 ^a^	9.43 ± 1.32 ^b^	13.27 ± 1.35 ^a^	8.77 ± 1.83 ^b^	0.0036
Total soluble proteins (7.70–9.00 ppm)	6.46 ± 0.340 ^a^	4.13 ± 0.63 ^b^	6.29 ± 0.58 ^a^	3.73 ± 0.88 ^b^	0.0021
Newly formulated feed					
	NT	Brine	PEF	PEF + Brine	*p*-value
Hydrophobic amino acids (0.20–2.00 ppm)	136.68 ± 6.84 ^a^	101.98 ± 5.25 ^b^	139.55 ± 13.59 ^a^	97.05 ± 2.01 ^b^	0.0003
Hydrophilic amino acids (2.00–3.00 ppm)	47.93 ± 2.34 ^a^	33.80 ± 1.70 ^b^	48.64 ± 5.27 ^a^	32.55 ± 1.06 ^b^	0.0002
Total amino acids (α protons) (3.20–4.70 ppm)	91.27 ± 4.15 ^a^	67.05 ± 3.4 ^b^	91.64 ± 6.45 ^a^	64.33 ± 2.30 ^b^	<0.0001
Aromatic amino acids (6.40–7.70 ppm)	12.62 ± 0.30 ^a^	8.53 ± 0.11 ^b^	12.78 ± 1.14 ^a^	8.88 ± 0.71 ^b^	0.0002
Total soluble proteins (7.70–9.00 ppm)	5.95 ± 0.20 ^a^	3.89 ± 0.22 ^b^	5.96 ± 0.51 ^a^	3.74 ± 0.24 ^b^	<0.0001

Data are means ± SD of 3 biological replicates. Statistical analysis was performed using one-way ANOVA with Tukey’s post hoc test. Different letters in the same row indicate statistical significance (at least *p* < 0.05). NT = non-treated; PEF = pulsed electric field.

**Table 10:** Protein solubility in NT and treated samples before in vitro digestion.

		Standard Feed		
NT	Brine	PEF	Brine + PEF	*p*-value
85.4 ± 10.1 ^a^	28.9 ± 4.8 ^b^	77.4 ± 10.0 ^a^	30.5 ± 6.1 ^b^	≤0.001
		Newly formulated feed		
NT	Brine	PEF	Brine + PEF	*p*-value
76.6 ± 13.1 ^b^	28.3 ± 4.5 ^c^	93.1 ± 10.6 ^a^	29.9 ± 4.4 ^c^	≤0.001

Data are expressed as mg protein/g sample and are means ± SD of 10 biological replicates. Statistical analysis was performed using one-way ANOVA with Tukey’s post hoc test. Different letters in the same row indicate statistical significance (at least *p* < 0.05). NT = non-treated; PEF = pulsed electric field.

## Data Availability

Data is contained within the article or Supplementary Materials.

## Supplementary Materials

The following supporting information can be downloaded at: https://www.mdpi.com/article/10.3390/foods12162991/s1, Figure S1: Sea bass liver and anterior gut histopathological examination; Table S1: Percentage fatty acid composition of non-digested NT and treated fillets from sea bass fed the different diets; Table S2: Percentage fatty acid composition of digested NT and treated fillets from sea bass fed the different diets.
