# Supplementary material for: Toward Sustainable and Healthy Fish Products—The Role of Feeding and Preservation Techniques
Source: Foods. 2023 Aug 8;12(16):2991. doi: 10.3390/foods12162991 (PMC10453833; doi:10.3390/foods12162991)
Supplement: Supplementary file 1 [file foods-12-02991-s001.zip › Antonelli et al_Supplementary figure S1.pdf]

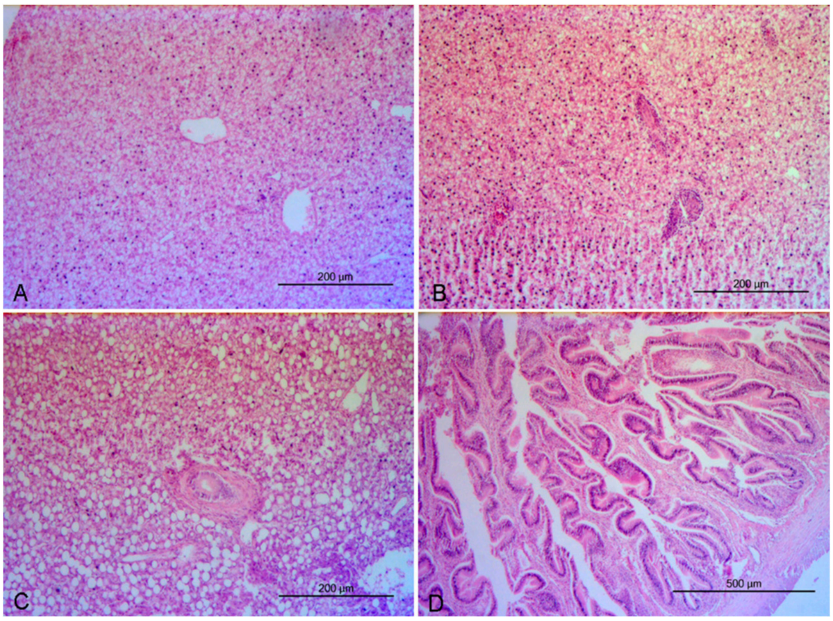

Figure S1. Sea bass liver and anterior gut histopathological examination. A) Mild lipid accumulation. B) Mild lipid accumulation and white blood cells infiltration C) Steatosis. D) Normal anterior gut.
