# Supplementary material for: Toward Sustainable and Healthy Fish Products—The Role of Feeding and Preservation Techniques
Source: Foods. 2023 Aug 8;12(16):2991. doi: 10.3390/foods12162991 (PMC10453833; doi:10.3390/foods12162991)
Supplement: Supplementary file 1 [file foods-12-02991-s001.zip › Antonelli et al_supplementary table S1_revised.pdf]

Table S1. Percent fatty acids composition of not-digested NT and treated fillets from seabass fed the different diets. Data are expressed as mol/100 mol FAMEs and are means ± SD of 3 biological replicates. Statistical analysis was the one-way ANOVA using Tukey's as post-hoc test. Different letters in the same row indicate statistical significance (at least p<0.05). NT = not treated.

**Standard feed**					
	NT	Brining	PEF	Brining + PEF	p value
14:0	3.31 ± 0.07[a]	2.64 ± 0.24[b]	3.21 ± 0.36[ab]	3.46 ± 0.35[a]	0.0322
16:0	19.78 ± 0.44[a]	21.04 ± 0.86[a]	19.97 ± 0.48[a]	20.16 ± 0.38[a]	0.1052
16:1 n-7	3.98 ± 0.28[a]	3.71 ± 0.26[a]	3.99 ± 0.21[a]	4.75 ± 0.35[b]	0.0096
18:0	4.15 ± 0.26[a]	5.15 ± 0.06[b]	4.25 ± 0.28[a]	4.23 ± 0.35[a]	0.0047
18:1 n-9	25.82 ± 0.78[a]	24.25 ± 0.91[a]	26.14 ± 1.51[a]	26.04 ± 1.18[a]	0.2180
18:2 n-6	9.69 ± 0.05[a]	8.42 ± 0.10[b]	9.63 ± 0.26[a]	9.74 ± 0.57[a]	0.0025
18:3 n-3	2.40 ± 0.10[a]	2.07 ± 0.04[b]	2.52 ± 0.13[a]	2.48 ± 0.12[a]	0.0027
20:1 n-9	5.69±0.13[a]	4.89±0.58[a]	5.34 ± 0.81[a]	5.97 ± 0.25[a]	0.1401
20:4 n-6	0.98 ± 0.01[a]	1.19 ± 0.11[b]	1.05 ± 0.02[ab]	0.97 ± 0.09[a]	0.0194
20:5 n-3	6.78 ± 0.47[a]	6.78 ± 0.39[a]	6.71 ± 0.14[a]	6.29 ± 0.30[a]	0.3170
22:5 n-3	1.49±0.04[a]	1.42 ± 0.07[a]	1.46 ± 0.02 [a]	1.40 ± 0.04[a]	0.1547
22:6 n-3	15.92 ± 0.73[a]	18.43±2.11[a]	15.72 ± 0.37[a]	14.52 ± 1.72[a]	0.0517
**New formulated feed**					
	NT	Brining	PEF	Brining + PEF	p value
14:0	3.12 ± 0.12[a]	2.84 ± 0.20[a]	2.88 ± 0.34[a]	2.89 ± 0.27[a]	0.5303
16:0	20.37 ± 0.21[a]	20.36 ± 0.85[a]	20.10 ± 0.64[a]	20.54 ± 0.32[a]	0.7287
16:1 n-7	4.42 ± 0.10[a]	4.11 ± 0.14[a]	4.30 ± 0.26[a]	4.22 ± 0.09[a]	0.2005
18:0	4.47 ± 0.15[a]	5.00 ± 0.19[a]	4.61 ± 0.55[a]	4.90 ± 0.25[a]	0.2385
18:1 n-9	30.14 ± 0.37[a]	28.48 ± 0.47[a]	29.03 ± 1.03[a]	29.10 ± 0.46[a]	0.0681
18:2 n-6	9.88 ± 0.24[a]	9.63 ± 0.28[a]	9.64 ± 0.41[a]	9.57 ± 0.30[a]	0.6500
18:3 n-3	2.44 ± 0.06[a]	2.43 ± 0.09[a]	2.42 ± 0.11[a]	2.48 ± 0.14[a]	0.8994
20:1 n-9	4.85 ± 0.19[a]	4.69 ± 0.34[a]	4.65 ± 0.22[a]	4.86 ± 0.04[a]	0.5803
20:4 n-6	0.91 ± 0.03[a]	1.04 ± 0.05[b]	1.01 ± 0.06[ab]	1.01 ± 0.02[ab]	0.0273
20:5 n-3	5.69 ± 0.03[a]	6.22 ± 0.51[a]	6.18 ± 0.48[a]	5.85 ± 0.11[a]	0.3320
22:5 n-3	1.35 ± 0.04[a]	1.30 ± 0.04[a]	1.36 ± 0.09[a]	1.31 ± 0.02[a]	0.4872
22:6 n-3	12.36 ± 0.50[a]	13.90 ± 1.10[a]	13.83 ± 1.34[a]	13.27 ± 0.02[a]	0.2182
