# Supplementary material for: Toward Sustainable and Healthy Fish Products—The Role of Feeding and Preservation Techniques
Source: Foods. 2023 Aug 8;12(16):2991. doi: 10.3390/foods12162991 (PMC10453833; doi:10.3390/foods12162991)
Supplement: Supplementary file 1 [file foods-12-02991-s001.zip › Antonelli et al_Supplementary table S2_revised.pdf]

Table S2. Percent fatty acids composition of digested NT and treated fillets from seabass fed the different diets. Data are expressed as mol/100 mol FAMEs and are means ± SD of 3 biological replicates. Statistical analysis was the one-way ANOVA using Tukey's as post-hoc test. Different letters in the same row indicate statistical significance (at least $p<0.05$). NT = not treated

	NT	Brining	PEF	Brining + PEF	p value
**Standard feed**					
14:0	3.40 ± 0.03^{a}	3.41 ± 0.05^{a}	3.65 ± 0.30^{a}	3.65 ± 0.07^{a}	0.1388
16:0	21.12 ± 0.44^{a}	21.45 ± 0.68^{a}	20.78 ± 0.54^{a}	20.46 ± 0.50^{a}	0.2210
16:1 n-7	4.23 ± 0.27^{a}	4.25 ± 0.08^{a}	4.32 ± 0.39^{a}	4.31 ± 0.24^{a}	0.9683
18:0	4.25 ± 0.25^{a}	2.83 ± 0.32^{bc}	3.76 ± 0.34^{ab}	2.14 ± 0.55^{c}	0.0006
18:1 n-9	26.93 ± 0.47^{a}	27.58 ± 1.95^{a}	25.91 ± 0.29^{a}	26.95 ± 1.35^{a}	0.4545
18:2 n-6	9.47 ± 0.15^{a}	9.55 ± 0.21^{a}	9.76 ± 0.10^{a}	9.81 ± 0.40^{a}	0.3243
18:3 n-3	2.27 ± 0.10^{a}	2.43 ± 0.11^{a}	2.35 ± 0.03^{a}	2.51 ± 0.13^{a}	0.0832
20:1 n-9	6.16 ± 0.21^{a}	6.42 ± 0.40^{ab}	6.70 ± 0.14^{ab}	7.07 ± 0.23^{b}	0.0148
20:4 n-6	0.93 ± 0.02^{a}	0.94 ± 0.15^{a}	0.93 ± 0.07^{a}	0.83 ± 0.06^{a}	0.4300
20:5 n-3	6.13 ± 0.24^{a}	5.60 ± 0.22^{b}	6.36 ± 0.15^{a}	5.96 ± 0.36^{ab}	0.0344
22:5 n-3	1.38 ± 0.03^{a}	1.42 ± 0.13^{a}	1.39 ± 0.07^{a}	1.46 ± 0.10^{a}	0.7089
22:6 n-3	13.75 ± 0.69^{a}	14.13 ± 1.19^{a}	14.09 ± 0.84^{a}	14.85 ± 2.02^{a}	0.7672
**New formulated feed**					
	NT	Brining	PEF	Brining + PEF	p value
14:0	3.31 ± 0.22^{a}	3.24 ± 0.17^{a}	3.45 ± 0.05^{a}	3.53 ± 0.16^{a}	0.1973
16:0	21.24 ± 0.17^{a}	20.18 ± 0.56^{a}	20.94 ± 0.41^{a}	20.48 ± 0.73^{a}	0.1281
16:1 n-7	4.45 ± 0.34^{a}	4.53 ± 0.15^{a}	4.59 ± 0.02^{a}	4.77 ± 0.10^{a}	0.2881
18:0	4.51 ± 0.02^{a}	3.93 ± 0.20^{a}	4.01 ± 0.14^{a}	3.54 ± 0.83^{a}	0.1292
18:1 n-9	30.44 ± 0.29^{a}	31.26 ± 1.26^{a}	30.39 ± 0.36^{a}	31.62 ± 0.35^{a}	0.1516
18:2 n-6	9.83 ± 0.23^{a}	10.28 ± 0.10^{a}	9.93 ± 0.29^{a}	10.24 ± 0.37^{a}	0.1764
18:3 n-3	2.24 ± 0.04^{a}	2.49 ± 0.17^{a}	2.33 ± 0.05^{a}	2.42 ± 0.06^{a}	0.0559
20:1 n-9	5.13 ± 0.08^{a}	5.64 ± 0.19^{b}	5.32 ± 0.13^{ab}	5.61 ± 0.07^{b}	0.0032
20:4 n-6	0.87 ± 0.07^{a}	0.89 ± 0.07^{a}	0.88 ± 0.06^{a}	0.85 ± 0.09^{a}	0.9184
20:5 n-3	5.28 ± 0.23^{a}	5.35 ± 0.31^{a}	5.44 ± 0.07^{a}	5.10 ± 0.41^{a}	0.5406
22:5 n-3	1.38 ± 0.07^{a}	1.34 ± 0.08^{a}	1.37 ± 0.04^{a}	1.37 ± 0.12^{a}	0.9384
22:6 n-3	11.32 ± 0.81^{a}	10.87 ± 0.73^{a}	11.36 ± 0.38^{a}	10.47 ± 1.47^{a}	0.6292
